# Sexually transmitted infections and risk of epithelial ovarian cancer: results from the Nurses’ Health Studies

**DOI:** 10.1038/s41416-019-0422-9

**Published:** 2019-03-21

**Authors:** Renée T. Fortner, Kathryn L. Terry, Noemi Bender, Nicole Brenner, Katrin Hufnagel, Julia Butt, Tim Waterboer, Shelley S. Tworoger

**Affiliations:** 1 Hormones and Cancer Group, Division of Cancer Epidemiology, German Cancer Research Center (DKFZ), Heidelberg, Germany; 2 Ob/Gyn Epidemiology Center, Brigham and Women’s Hospital, Boston, MA USA; 3 Obstetrics, Gynecology and Reproductive Biology, Harvard Medical School, Boston, MA USA; 4 Department of Epidemiology, Harvard T.H. Chan School of Public Health, Boston, MA USA; 5 Infections and Cancer Epidemiology, Division of Molecular Diagnostics of Oncogenic Infections, German Cancer Research Center (DKFZ), Heidelberg, Germany; 6 Department of Cancer Epidemiology, Moffitt Cancer Center, Tampa, FL USA

**Keywords:** Gynaecological cancer, Risk factors

## Abstract

**Background:**

Sexually transmitted infections (STIs) are associated with pelvic inflammatory disease and tubal pathologies. Given the tubal origin of a proportion of ovarian cancers, STIs may be relevant in their aetiology.

**Methods:**

Antibodies indicating past infection with *Chlamydia trachomatis*, *Mycoplasma genitalium,* herpes simplex virus type 2, and against human papillomavirus oncogenes (L1 and E6+E7 oncoproteins of types 16, 18, 45) were measured in prediagnosis plasma samples in a nested case–control study in the Nurses’ Health Studies (*n* = 337 cases 1:1 matched to controls). Logistic regression was used to estimate multivariable-adjusted relative risks (RRs) and 95% confidence intervals [CIs] comparing women seropositive vs. seronegative among all cases (invasive and borderline), invasive (*n* = 257), and invasive serous ovarian cancers; *n* = 170), and borderline ovarian tumours (*n* = 80).

**Results:**

*C. trachomatis* seropositivity was associated with higher risk of ovarian cancer overall (RR = 2.07 [1.25–3.43]); results were similar for invasive, invasive serous, and borderline tumours. We observed no associations for the other STIs. Relative to women seronegative to all infections, strongest associations were observed for seropositivity to *C. trachomatis* plus another STI (2.74 [1.20–6.27]; *C. trachomatis* alone, 1.88 [1.03–3.42]; all cases); however, the RRs were not significantly different.

**Conclusions:**

*C. trachomatis* infection may increase ovarian cancer risk; additional studies are required.

## Background

Sexually transmitted infections (STIs), including bacterial infections such as *Chlamydia trachomatis* and *Mycoplasma genitalium* and viral infections like human papillomavirus (HPV) and herpes simplex virus type 2 (HSV-2), can induce persistent changes in the female genital tract. *C. trachomatis* and *M. genitalium* are associated with pelvic inflammatory disease (PID), salpingitis, and tubal infertility.^1–3^ PID was associated with increased ovarian cancer risk in a recent meta-analysis,^4^ providing indirect evidence for a role for STIs in the aetiology of the disease. STI-induced tubal pathologies may be relevant given that a proportion of ovarian tumours likely originate in the fallopian tube,^5,6^ with tubal involvement or precursor serous tubal intraepithelial carcinomas (STICs) observed in up to 70% of high-grade serous ovarian cancers.^7^

Prior retrospective studies, and a single prospective study, on *C. trachomatis* and ovarian cancer risk are suggestive of an association^8–12^; data on other STIs are sparse.^12^ Infection with mucosal high-risk types of HPV (e.g. 16, 18) is recognised as a cause of cervical and other anogenital malignancies.^13^ Incidence of STIs is rising in many developed countries, with 1.6 million reported cases of *C. trachomatis* in the United States in 2016.^14^ There are relatively few modifiable risk factors for ovarian cancer; STI prevention would provide a target for primary prevention of this often-lethal disease. To investigate the association between STIs and ovarian cancer risk, we conducted a nested case–control study on seropositivity to *C. trachomatis* and *M. genitalium*, HSV-2, and multiple proteins of HPV16, HPV18, and HPV45, with risk of borderline ovarian tumours (BOT) and invasive epithelial ovarian cancer (iEOC) in the Nurses’ Health Study (NHS) and NHSII among 337 cases and 337 matched controls.

## Materials and methods

### Study population and biospecimen collection

The NHS was initiated in 1976 when 121,700 registered nurses, ages 30–55 years, completed and returned a mailed questionnaire.^15^ The NHSII began in 1989 with 116,429 female registered nurses ages 25–42 years, using the same approach. Participants in both cohorts have been followed biennially to update information on lifestyle factors and ascertain disease diagnoses.

In 1989–1990, 32,826 NHS participants provided a blood sample (ages 43–70 years); in 2000–2002, a subset of 18,743 of these participants (ages 53–80 years) provided a second sample. Between 1996 and 1999, 29,611 NHSII participants (ages 32–54 years) provided blood samples.^16^ A second collection among 15,982 women (ages 44–68 years) was carried out between 2008 and 2011. Similar methods were used for all collections.^17–19^ Heparin plasma samples have been stored in liquid nitrogen freezers since collection. This study was approved by the Institutional Review Board of the Brigham and Women’s Hospital (Boston, MA).

### Case and control selection

Eligible cases were diagnosed with confirmed incident ovarian cancer (i.e. BOT or iEOC) after first blood collection. A total of 337 cases (NHS, *n* = 271; NHSII, *n* = 66) were diagnosed through June 1, 2016 (NHS) and June 1, 2015 (NHSII). Cases were matched to one control, who was alive and had intact ovaries at the time of case diagnosis (see Supplemental Methods). For participants with two prediagnosis blood samples available, the sample proximate to diagnosis (or selection as a control) was analysed.

### Laboratory analyses

Plasma samples were tested for antibodies using a multiplex, fluorescent bead-based assay (see Supplemental Methods).^20^ Women were defined as *C. trachomatis* seropositive when positive for the Pgp3 antibody.^12^ Past/current *M. genitalium* infection was evaluated using antibodies to MgPa N-Terminus and rMgPa.^12^ HSV-2 was assessed evaluating antibodies to 2mgG unique.^21^ HPV infection with HPV types 16, 18, and 45 was assessed evaluating antibodies to the corresponding L1, E6, and E7 proteins. Given the low prevalence of individual types, HPV positive was defined as positive to any of the following: HPV16 E6, which has been shown to be a stand-alone marker for higher risk of HPV16-associated oropharyngeal cancer,^22^ or HPV18 E6 and E7 or HPV45 E6 and E7 as the combination of E6 and E7 increases specificity for cervical cancer.^23^ In a secondary analysis, seropositivity to HPV L1 proteins of HPV16, 18, or 45 was evaluated.

In addition to evaluating the individual infections, we compared women seropositive for *C. trachomatis* plus any other STI to women seronegative for all STIs. In a secondary analysis, we dichotomised women seropositive for *C. trachomatis* by the laboratory cut point (200 mean fluorescent intensity (MFI)) into subgroups with higher vs. lower antibody levels using the median in all *C. trachomatis* positive women (2668 MFI) as the cut point.

### Statistical analyses

Conditional logistic regression was used to estimate relative risks (RRs) and 95% confidence intervals [CIs] for ovarian cancer overall. Unconditional logistic regression, adjusted for the matching factors, was used in analyses restricted to BOT, iEOC, and serous iEOC; there were too few cases of other histotypes (e.g. endometrioid, clear cell) to assess separately (*n* = 53). We evaluated heterogeneity by rapidly fatal disease (died within 3 years of diagnosis) and less aggressive disease (survived at least 3 years following diagnosis) comparing models allowing vs. not allowing the association for the STI to vary by disease aggressiveness using a likelihood ratio test.^24^

Multivariable models were adjusted for parity (nulliparous, 1, 2, 3, 4+ pregnancies), oral contraceptive (OC) use (never, <1 year, 1–5 years, 5+ years), tubal ligation (yes, no), marital status (never, married/domestic partnership/living with partner, divorced/separated, widowed), and weight change between ages 18 and blood collection (kg, continuous); these variables were selected a priori based on their association with ovarian cancer or STI prevalence. Additional adjustment for family history of breast and/or ovarian cancer, non-steroidal anti-inflammatory or aspirin use (never, <5 year, 5–9 year, 10–14 year, 15+ year duration), or depressive symptoms (Mental Health Index: ≤52 vs, >52) did not change effect estimates by ≥10%.

Potential effect modification by menopausal status at blood collection and diagnosis (premenopausal vs. postmenopausal), age at blood collection (<60 vs. 60+ years), and ever OC use was evaluated by including interaction terms and evaluating the Wald test. Women reporting tubal ligation (*n* = 127) or who were nulliparous (*n* = 84) were excluded in sensitivity analyses. Statistical analyses were conducted in SAS 9.4 (Cary, NC). All statistical tests were two sided, and *p* < 0.05 was considered statistically significant.

## Results

A total of 337 cases matched to 337 controls were included in this study. Participants were median age of 60 years at blood collection (range: 34–81) and predominantly postmenopausal (69%). Cases were diagnosed at median age of 68 years (range: 37–89); 76% were diagnosed with invasive disease (*n* = 257), and 66% of invasive cases were of serous histology (*n* = 170) (Table 1).

**Table 1:** Baseline characteristics of study participants (*n* (%) or median (range)): results from the NHS and NHSII

	Cases	Controls
	*n* = 337	*n* = 337
Age at blood collection, years	60 (34 to 81)	60 (35 to 80)
Menopausal status at blood collection		
Premenopausal	77 (23)	76 (23)
Postmenopausal	232 (69)	232 (69)
Perimenopausal/unknown	28 (9)	29 (9)
Parity		
Nulliparous	56 (17)	28 (8)
1	25 (7)	16 (5)
2	109 (32)	103 (31)
3	74 (22)	91 (27)
4+	73 (22)	99 (29)
OC use		
Never	158 (47)	160 (47)
<1 year	43 (13)	39 (12)
1–5 years	81 (24)	63 (19)
5+ years	55 (16)	75 (22)
Reported tubal ligation	53 (16)	74 (22)
Marital status		
Never married	10 (3)	4 (1)
Married/living with partner	268 (79)	273 (81)
Divorced/separated	20 (6)	26 (8)
Widowed	39 (12)	34 (10)
Weight change between age 18 years and blood collection, kg	11 (−30 to 91)	9 (−17 to 61)

Case characteristics		
Time between blood collection and diagnosis, years	7 (<1 to 25)	
Age at diagnosis, years	68 (37 to 89)	
Borderline ovarian tumours	80 (24)	
Invasive epithelial ovarian cancer	257 (76)	
Serous	170 (66)	
Non-serous^a^	53 (21)	
Rapidly fatal^b^	94 (37)	
Less aggressive disease^b^	144 (56)	

*NHS* Nurses' Health Study, OC oral contraceptive

^a^Non-serous: mucinous, endometrioid, and clear cell subtypes. ^b^Ovarian cancer death within 3 years of diagnosis/lived at least 3 years; restricted to women with at least 3 years of follow-up after diagnosis

Seropositivity to at least one STI was observed in 25% of the study population; seropositivity to *C. trachomatis* was the most common (20% cases; 12% controls) (Table S1). Eight percent of cases and 4% of controls were positive for more than one infection. The most frequently observed combination was *C. trachomatis* and HSV-2 (6% cases; 2% controls).

Seropositivity to *C. trachomatis* infection was associated with a two-fold increased risk of ovarian cancer (RR: 2.07 [95% CI: 1.15–3.43]); results were similar for invasive (1.98 [1.21–3.23]) and invasive serous disease (2.31 [1.33–4.01]), and BOT (2.11 [1.04–4.28]) (Table 2). Further, results were similar among *C. trachomatis* seropositive women regardless of antibody level (e.g. all cases, MFI below median 1.98 [1.05–3.76]; above median, 2.16 [1.11–2.41]; data not tabled). No significant association for antibodies to other individual infections and ovarian cancer risk was observed (*p* ≥ 0.11) including in the HPV L1 analyses (data not shown). *M. genitalium* had a suggestive positive association despite the low seroprevalence (1.92 [0.78–4.72]).

**Table 2:** Seropositivity to individual sexually transmitted infections and risk of ovarian cancer: results from the NHS and NHSII

	Control, *n*	All cases					Borderline ovarian tumours			Invasive EOC			Invasive serous EOC		
		Unadjusted			Adjusted^a^		Adjusted^a^			Adjusted^a^			Adjusted^a^		
		Case, *n*	RR	95% CI	RR	95% CI	Case, *n*	RR	95% CI	Case, *n*	RR	95% CI	Case, *n*	RR	95% CI
*C. trachomatis*															
Negative	297	271	Ref.		Ref.		63	Ref.		208	Ref.		135	Ref.	
Positive	40	66	2.04	1.26–3.29	2.07	1.25–3.43	17	2.11	1.04–4.28	49	1.98	1.21–3.23	35	2.31	1.33–4.01
*M. genitalium*															
Negative	328	320	Ref.		Ref.		74	Ref.		246	Ref.		164	Ref.	
Positive	9	17	2.00	0.86–4.67	1.92	0.78–4.72	6	2.75	0.86–8.76	11	1.81	0.68–4.77	6	1.59	0.51–4.98
Herpes simplex virus, type 2															
Negative	308	299	Ref.		Ref.		68	Ref.		231	Ref.		152	Ref.	
Positive	29	38	1.36	0.81–2.28	1.38	0.79–2.42	12	2.04	0.92–4.54	26	1.24	0.69–2.26	18	1.32	0.68–2.59
HPV16 E6 or HPV18 E6+E7 or HPV45 E6+E7															
Negative	330	326	Ref.		Ref.		77	Ref.		249	Ref.		164	Ref.	
Positive	7	11	1.57	0.61–4.05	1.23	0.44–3.44	3	2.03	0.47–8.77	8	1.37	0.47–3.99	6	1.84	0.58–5.88

*CI* confidence interval, *EOC* epithelial ovarian cancer, *HPV* human papillomavirus, *NHS* Nurses’ Health Study, *RR* relative risk

^a^Conditional logistic regression models for all cases; all other results are from unconditional logistic regression models additionally controlling for matching factors (year of birth (±1 year), menopausal status at diagnosis (premenopausal, postmenopausal, unknown), and factors at one or both blood draws: menopausal status (premenopausal, postmenopausal, unknown), month of collection (±1 month), time of day (±2 h), fasting status (>8, ≤8 h), and postmenopausal hormone use (yes/no). Premenopausal NHSII cases and controls additionally matched on luteal day at blood collection (date of next menstrual cycle minus date of blood draw, ±1 day)). All adjusted models adjusted for: parity (nulliparous, 1 pregnancy, 2 pregnancies, 3 pregnancies, 4+ pregnancies), oral contraceptive use (never, <1 year, 1–5 years, 5+ years), tubal ligation (yes, no), marital status (never, married/domestic partnership/living with partner, divorced/separated, widowed), and weight change between ages 18 years and blood collection (kg, continuous)

We next evaluated antibodies to *C. trachomatis* plus the other infections and ovarian cancer risk. Relative to women seronegative to all evaluated infections, seropositivity to *C. trachomatis* and any other infection was associated with a 2.74-fold higher risk of ovarian cancer (95% CI: 1.20–6.27), whereas *C. trachomatis* alone was associated with a 1.88-fold higher risk (95% CI: 1.03–3.42) (Table 3). Considering the combinations of antibodies to individual infections, seropositivity to both *C. trachomatis* and *M. genitalium* and *C. trachomatis* and HSV-2 were associated with higher risk than seropositivity to *C. trachomatis* alone. However, these differences were not statistically significant (*p* ≥ 0.14).

**Table 3:** Cross-classification of seropositivity to *C. trachomatis* and other sexually transmitted infections and risk of ovarian cancer: results from the NHS and NHSII

	Control, *n*	All cases			Invasive EOC		
		Case, *n*	RR^a^	95% CI	Case, *n*	RR^a^	95% CI
*C. trachomatis* (CT) and *M. genitalium* (MG)							
CT− and MG−	291	263	Ref.		203	Ref.	
CT+ and MG−	37	57	1.89	1.11–3.20	43	1.79	1.08–2.97
CT− and MG+	6	8	1.12	0.34–3.74	5	0.93	0.26–3.31
CT+ and MG+	3	9	4.38	1.03–18.56	6	5.74	1.23–26.72
CT and herpes simplex virus, type 2 (HSV2)							
CT− and HSV2−	276	253	Ref.		197	Ref.	
CT+and HSV2−	32	46	1.85	1.06–3.24	34	1.67	0.97–2.87
CT− and HSV2+	21	18	1.01	0.50–2.03	11	0.73	0.33–1.62
CT+ and HSV2+	8	20	2.90	1.14–7.37	15	3.26	1.25–8.51
CT and HSV and MG							
CT− and HSV2− and MG−	272	245	Ref.		192	Ref.	
CT+ and HSV2− and MG−	30	41	1.77	0.98–3.18	32	1.64	0.94–2.85
CT− and (HSV2+ or MG+)	25	26	1.19	0.63–2.23	16	0.90	0.45–1.81
CT+ and (HSV2+ or MG+)	10	25	3.04	1.29–7.21	17	3.25	1.33–7.96
CT and HSV and MG and human papillomavirus (HPV) 16 E6, HPV18 E6+E7, or HPV45 E6+E7							
Negative for all	270	237	Ref.		186	Ref.	
CT+ only	29	41	1.88	1.03–3.42	32	1.73	0.99–3.03
MG+, HSV2+ or HPV+ only	27	34	1.34	0.80–2.24	22	1.19	0.64–2.24
CT+ and any other infection	11	25	2.74	1.20–6.27	17	3.01	1.26–7.18

CT and HPV combination not evaluated, 0 cases and 3 controls positive for both infections

*CI* confidence interval, *EOC* epithelial ovarian cancer, *NHS* Nurses’ Health Study, *RR* relative risk

^a^Conditional logistic regression models for all cases; all other results are from unconditional logistic regression models additionally controlling for matching factors (year of birth (±1 year), menopausal status at diagnosis (premenopausal, postmenopausal, unknown), and factors at one or both blood draws: menopausal status (premenopausal, postmenopausal, unknown), month of collection (±1 month), time of day (±2 h), fasting status (>8, ≤8 h), and postmenopausal hormone use (yes/no). Premenopausal NHSII cases and controls additionally matched on luteal day at blood collection (date of next menstrual cycle minus date of blood draw, ±1 day)). All adjusted models adjusted for: parity (nulliparous, 1 pregnancy, 2 pregnancies, 3 pregnancies, 4 + pregnancies), oral contraceptive use (never, <1 year, 1–5 years, 5+ years), tubal ligation (yes, no), marital status (never, married/domestic partnership/living with partner, divorced/separated, widowed), and weight change between ages 18 years and blood collection (kg, continuous)

Associations excluding women with a tubal ligation were generally similar (e.g. *C. trachomatis*, RR: 1.94 [1.17–3.21]; Table S2). Associations were similar in analyses restricted to parous women, with the exception of a significant positive association observed between *M. genitalium* and ovarian cancer risk (RR: 3.40 [1.25–9.27]). We observed no significant differences in the associations by menopausal status at blood collection or diagnosis, age at blood collection, or ever OC use. Further, the associations did not differ significantly for rapidly fatal and less aggressive disease (*p* ≥ 0.18).

## Discussion

This study suggests that past/current infection with *C. trachomatis*, a common STI, may be associated with subsequent ovarian cancer risk, with a potentially synergistic effect of infection with *C. trachomatis* together with other STIs (e.g. *M. genitalium* or HSV-2), though effect estimates were not significantly different. Associations were similar or slightly stronger when restricting to cases with invasive serous disease; this association is notable given that relatively few risk factors have been identified for this subtype. Our associations were robust across different subsets of women, including those with a prior tubal ligation and parous women.

*C. trachomatis* may directly affect ovarian carcinogenesis as it induces DNA double-strand breaks, interferes with the DNA damage response,^25^ and inhibits apoptosis in the host cell.^26^ Importantly, *C. trachomatis* and *M. genitalium* in particular may indirectly influence ovarian cancer development through increasing inflammation in the genital tract. Specifically, these infections are an important cause of salpingitis and other tubal pathologies,^27^ including tubal damage-inducing PID.^1,28^ This may be particularly relevant for the serous histotype, as the fallopian tube has been identified as the origin of the majority of these tumours,^6^ with STICs as a suggested precursor lesion.^29^ Inflammation, and inflammation-related sequelae, are likely major mechanisms linking STIs and PID to higher ovarian cancer risk, and inflammation^30,31^ and its downstream impact on immune suppression may play a role in risk of ovarian cancer regardless of subtype. A recent meta-analysis of 13 studies reported a significant association between history of PID infection and ovarian cancer risk (RR: 1.25 [1.06–1.44]), though this association was limited to borderline disease (invasive, RR: 1.15 [0.89–1.49]; borderline, RR: 1.42 [1.25–1.63]), and no histology-specific estimates were provided.^4^ Price et al. recently estimated probabilities ranging from 12% to 16% of *C. trachomatis* infection leading to clinical PID.^32^ No data on PID or self-reported infection status were available in our study; thus we could not investigate this as a potential mediator.

Retrospective data on history of chlamydia infection and ovarian cancer risk^8–11^ have assessed different IgG antibodies (e.g. heat shock protein 60—type 1 (cHSP60-1),^8^ elementary bodies of serovar D^9,10^). Results from these studies are mixed, in part, because the antibodies have higher cross-reactivity with other chlamydial and bacterial infections than the gold standard Pgp3-based antibody. Further, it is unclear whether having ovarian cancer or being under treatment may alter the expression of IgG antibodies. More recent data from Trabert et al. using the same laboratory methods as this study reported a positive association between antibodies to *C. trachomatis* infection and iEOC risk in a population-based case–control study in Poland (*n* = 244 cases) and in the Prostate, Lung, Colorectal, and Ovarian (PLCO) Cancer Screening Trial (*n* = 159 cases).^12^ In the latter study (as in our study), blood samples were collected prior to diagnosis of ovarian cancer, reducing impact of disease status (i.e. prevalent iEOC) on immune functioning.

In the PLCO study, a significant association was only observed when a higher cut point was used to define Pgp3 positivity (laboratory cut point, RR = 1.43 [0.78–2.63]); elevated cut point based on Poland study, RR = 2.25 [1.07–4.71]), whereas in the current study, a two-fold increase in risk was observed using the laboratory cut point (RR = 2.07 [1.25–3.43]). This may, in part, be due to the larger sample size in our study or underlying differences in the study populations (e.g. 12% of controls in the current study vs. 21.4% of controls in the PLCO were positive for *C. trachomatis* using the laboratory cut point). In the current study, we did not observe that, among seropositive women, higher titres of *C. trachomatis* antibody levels were more strongly associated with risk. While higher antibody levels may be associated with severity or number of past infections or with more severe tubal damage,^33^ antibody levels in response to an infection are a function of many other factors including age at infection and individual-level immune response. Further research is needed to better understand how antibody titres relate to disease severity and carcinogenic-related damage and inflammation to the fallopian tubes and ovaries.

We observed no significant association between antibodies to *M. genitalium*; HSV-2; or HPV types 16, 18, and 45 and ovarian cancer risk, although the results were suggestive of an association for *M. genitalium*, which had a low prevalence in our population. *M. genitalium* and HSV-2 induce alterations (e.g. oedema, malformed cilia) in the fallopian tube following infection.^34,35^ *M. genitalium* seropositivity was associated with borderline, but not invasive, tumours in one small study,^8^ and the expression of viral microRNA, predominantly mapped to HSV-1 and HSV-2 genomes, was observed to be higher in serous ovarian carcinomas than in normal tissue.^36^ Trabert et al. observed a 47% higher risk of iEOC among women seropositive to *M. genitalium* in the Poland case–control study, but no association in PLCO; HSV-2 was also not related to risk in that study.^12^ *M. genitalium* is thought to be a cause of PID and warrants further study in larger populations with a higher seroprevalence. HPV oncoproteins promote genomic instability and proliferation and inhibit apoptosis,^37^ and high-risk HPV types are causally linked to cervical cancer, other anogenital cancers, and cancers of the head and neck.^13^ These infections have been minimally explored in relation to ovarian cancer but, in general, have not been related to ovarian cancer development,^8,12,38^ suggesting that HPV does not infect ovarian or fallopian tube cells.

The association between *C. trachomatis* and ovarian cancer risk appeared stronger when a woman was also seropositive to *M. genitalium* or HSV-2. This may reflect higher incidence of tubal pathologies following infection with multiple STIs or, alternatively, may be the sequelae of a constellation of other factors (e.g. age at first intercourse, number of sexual partners). A limitation of our study is the lack of data on covariates such as sexual history and history of PID and/or other pelvic diseases and their treatment, thus it is possible that our results are, in part, due to residual confounding by these factors. As no studies have examined the relationship of sexual history with ovarian cancer risk, it is unclear whether this may be an important confounder.

This study assessed antibodies indicating history of STI infection using a validated assay in blood samples collected median 7 years prior to diagnosis. Antibodies are relatively stable in blood, thus we expect misclassification of exposure to be minimal and non-differential. However, while we know that an infection was present at or at some time prior to blood collection, and thus diagnosis, we cannot assess when the infection initially occurred, or whether subsequent infections manifested in the interval between blood collection and diagnosis, or what proportion of women with negative serology had history of an infection but did not seroconvert. We expect these issues be similar among women who developed ovarian cancer after blood collection and women who remained cancer-free and thus would result in non-differential misclassification of the exposure.

In summary, in a large, prospective epidemiologic study, we observed an increased risk of ovarian cancer overall and of the serous histotype among women seropositive for *C. trachomatis* infection, with strongest associations observed among women seropositive to *C. trachomatis* and another STI (i.e. *M. genitalium*, HSV-2). Additional experimental studies to establish causality, epidemiologic studies including data on PID to investigate this potential mediator, and larger epidemiologic studies to evaluate disease subtype are needed to confirm these associations. If these results are confirmed, it would suggest that STI prevention and early detection efforts, including vaccine development, may represent an opportunity to reduce ovarian cancer risk.

## Supplementary information

Supplemental Materials and Methods

Supplemental Tables S1 and S2

## Data Availability

Information on gaining access to NHS/NHSII resources is available at: http://www.nurseshealthstudy.org/researchers. Permission to access the data was granted by the Nurses’ Health Study leadership.
