# Supplementary material for: Sexually transmitted infections and risk of epithelial ovarian cancer: results from the Nurses’ Health Studies
Source: Br J Cancer. 2019 Mar 21;120(8):855–60. doi: 10.1038/s41416-019-0422-9 (PMC6474309; doi:10.1038/s41416-019-0422-9)
Supplement: Supplementary file 1 — Supplemental Materials and Methods [file 41416_2019_422_MOESM1_ESM.docx]

**Supplemental Methods**

*Case and Control Selection*

Cases and controls were matched on year of birth (+/-1 year), menopausal status at diagnosis (premenopausal, postmenopausal, unknown) and factors at one or both blood draws: menopausal status (premenopausal, postmenopausal, unknown), month of collection (+/-1 month), time of day (+/-2 hours), fasting status (>8, ≤8 hours), and postmenopausal hormone use (yes/no). For premenopausal NHSII cases, we additionally matched on luteal day (date of next menstrual cycle minus date of blood draw, +/- 1 day).

*Laboratory Analyses*

Viral and bacterial protein antigens were recombinantly expressed as glutathione S-transferase (GST) fusion proteins in *E.coli*, and loaded onto glutathione casein-coated fluorescence-labelled polystyrene beads (Luminex Corp. Austin, Texas, USA) by *in situ* affinity purification from crude bacterial lysate. Loading each antigen onto specific bead sets distinguishable by the internal fluorescence allows simultaneous measurement of antibodies against different antigens within one reaction vessel. Detection of bound primary antibodies from serum took place with a biotinylated goat-α-human IgM/IgG/IgA secondary antibody and subsequent incubation with fluorescent reporter conjugate streptavidin-R-phycoerythrin. Antibodies bound to each bead set were quantified at a 1:100 serum dilution as median fluorescence intensity (MFI). Continuous MFI values representing antibody levels were used to dichotomized as seropositive or seronegative based on previously defined cutpoints.[^1^](#_ENREF_1)

For *C. trachomatis,* serum antibodies to major outer membrane proteins (MOMP) from serovars A, D, and L2, translocated actin-recruiting protein N and C terminal fragments (Tarp-F1 and Tarp-F2), heat shock protein 60 variant 1 (HSP60-1),[^2^](#_ENREF_2) and plasmid-encoded Pgp3 protein were measured.[^1^](#_ENREF_1) The C. trachomatis Pgp3 protein is the gold standard due to longer persistence of antibodies compared with other antigens and higher sensitivity at high specificity.[^3^](#_ENREF_3)^,^ [^4^](#_ENREF_4) Here, 93% of women positive using a combination of the *C. trachomatis* antibodies (≥3 positive) were positive for the Pgp3 antibody.

**References**

1. Trabert B, Waterboer T, Idahl A, Brenner N, Brinton LA, Butt J, et al. Antibodies Against Chlamydia trachomatis and Ovarian Cancer Risk in Two Independent Populations. (2018) J Natl Cancer Inst; PMID: 29790947.

2. Hulstein SH, Matser A, Alberts CJ, Snijder MB, Willhauck-Fleckenstein M, Hufnagel K, et al. Differences in Chlamydia trachomatis seroprevalence between ethnic groups cannot be fully explained by socioeconomic status, sexual healthcare seeking behavior or sexual risk behavior: a cross-sectional analysis in the HEalthy LIfe in an Urban Setting (HELIUS) study. (2018) BMC Infect Dis.18;1:612; PMID: 30509189; PMCID: PMC6278015.

3. Wills GS, Horner PJ, Reynolds R, Johnson AM, Muir DA, Brown DW, et al. Pgp3 antibody enzyme-linked immunosorbent assay, a sensitive and specific assay for seroepidemiological analysis of Chlamydia trachomatis infection. (2009) Clinical and vaccine immunology : CVI.16;6:835-43; PMID: 19357314; PMCID: 2691054.

4. Woodhall SC, Wills GS, Horner PJ, Craig R, Mindell JS, Murphy G, et al. Chlamydia trachomatis Pgp3 Antibody Population Seroprevalence before and during an Era of Widespread Opportunistic Chlamydia Screening in England (1994-2012). (2017) PLoS One.12;1:e0152810; PMID: 28129328; PMCID: 5271337 adherence to PLOS ONE policies on sharing data and materials.
