# Supplementary material for: Sexually transmitted infections and risk of epithelial ovarian cancer: results from the Nurses’ Health Studies
Source: Br J Cancer. 2019 Mar 21;120(8):855–60. doi: 10.1038/s41416-019-0422-9 (PMC6474309; doi:10.1038/s41416-019-0422-9)
Supplement: Supplementary file 2 — Supplemental Tables S1 and S2 [file 41416_2019_422_MOESM2_ESM.docx]

| **Table S1. Prevalence of Seropositivity to sexually transmitted infections (*n* (%)): Results from the NHS and NHSII** | | | |
| --- | --- | --- | --- |
|  | **Cases**  ***n*=337** |  | **Controls**  ***n*=337** |
| **Seropositivity to single infections** |  |  |  |
| *C. trachomatis* | 66 (20) |  | 40 (12) |
| *M. genitalium* | 17 (5) |  | 9 (3) |
| Herpes simplex virus, type 2 (HSV2) | 38 (11) |  | 29 (9) |
| HPV16 E6 | 11 (3) |  | 5 (1) |
| HPV18 E6+E7 | 0 (0) |  | 0 (0) |
| HPV45 E6+E7 | 1 (0.3) |  | 3 (1) |
| Any of the above HPV types* | 11 (3) |  | 7 (2) |
|  |  |  |  |
| **Seropositivity to *C. trachomatis* and other infections** |  |  |  |
| *C. trachomatis* alone | 41 (12) |  | 29 (9) |
| *+ M. genitalium* | 5 (1.5) |  | 2 (1) |
| *+* HSV2 | 16 (4.8) |  | 5 (1.5) |
| *+* any HPV* | 0 (0) |  | 1 (0.3) |
| *+* HSV2 + any HPV* | 0 (0) |  | 2 (1) |
| *+ M. genitalium* + HSV2 | 4 (1.2) |  | 1 (0.3) |
| *+ M. genitalium +* any HPV* | 0 (0) |  | 0 (0) |
| *** HPV16 E6, or HPV18 E6+E7, or HPV45 E6+E7 |  |  |  |

| **Table S2. Sexually transmitted infections and risk of ovarian cancer restricted to women with no reported tubal ligation*: Results from the NHS and NHSII** | | | | | | | | | | | | | | | | | |
| --- | --- | --- | --- | --- | --- | --- | --- | --- | --- | --- | --- | --- | --- | --- | --- | --- | --- |
|  |  | **All cases** | | | | |  | | **Invasive EOC** | | | |  | | **Serous Invasive EOC** | | |
|  | **control n** | **case n** |  | **RR^a^** | **95% CI** |  | | **case n** | | **RR^a^** | **95% CI** |  | | **case n** | | **RR^a^** | **95% CI** |
| ***C. trachomatis*** | |  |  |  |  |  | |  | |  |  |  | |  | |  |  |
| Negative | 230 | 225 |  | Ref | |  | | 176 | | ref | |  | | 112 | | ref | |
| Positive | 33 | 59 |  | 1.94 | 1.17-3.21 |  | | 42 | | 1.87 | 1.09-3.22 |  | | 29 | | 2.19 | 1.18-4.08 |
|  |  |  |  |  |  |  | |  | |  |  |  | |  | |  |  |
| ***M. genitalium*** |  |  |  |  |  |  | |  | |  |  |  | |  | |  |  |
| Negative | 256 | 269 |  | Ref | |  | | 209 | | ref | |  | | 137 | | ref | |
| Positive | 7 | 15 |  | 1.94 | 0.74-5.11 |  | | 9 | | 1.62 | 0.55-4.83 |  | | 4 | | 1.15 | 0.30-4.46 |
|  |  |  |  |  |  |  | |  | |  |  |  | |  | |  |  |
| **Herpes simplex virus, type 2** | | |  |  |  |  | |  | |  |  |  | |  | |  |  |
| Negative | 236 | 253 |  | ref | |  | | 198 | | ref | |  | | 128 | | ref | |
| Positive | 27 | 31 |  | 0.99 | 0.55-1.78 |  | | 20 | | 0.85 | 0.44-1.64 |  | | 13 | | 0.89 | 0.42-1.93 |
|  |  |  |  |  |  |  | |  | |  |  |  | |  | |  |  |
| **HPV16 E6, or HPV18 E6+E7, or HPV45 E6+E7** | | | | |  |  | |  | |  |  |  | |  | |  |  |
| Negative | 256 | 277 |  | ref | |  | | 212 | | Ref | |  | | 137 | | ref | |
| Positive | 7 | 7 |  | 0.77 | 0.25-2.40 |  | | 6 | | 0.87 | 0.27-2.85 |  | | 4 | | 1.23 | 0.32-4.74 |
|  |  |  |  |  |  |  | |  | |  |  |  | |  | |  |  |
| **Multiple Infections** | |  |  |  |  |  | |  | |  |  |  | |  | |  |  |
| negative for all** | 206 | 200 |  | Ref | |  | | 160 | | Ref | |  | | 101 | | ref | |
| CT only | 23 | 37 |  | 1.80 | 1.00-3.23 |  | | 28 | | 1.71 | 0.92-3.18 |  | | 21 | | 2.16 | 1.08-4.31 |
| MG, HSV2 or HPV only | 24 | 25 |  | 1.02 | 0.54-1.93 |  | | 16 | | 0.82 | 0.40-1.68 |  | | 11 | | 1.10 | 0.48-2.49 |
| CT+other | 10 | 22 |  | 2.33 | 1.00-5.43 |  | | 14 | | 2.19 | 0.85-5.61 |  | | 8 | | 2.43 | 0.77-7.66 |
| *127 participants (53 cases; 74 controls) excluded.  **^a^**Conditional logistic regression models for all cases; all other results are from unconditional logistic regression models controlling for matching factors ( year of birth (+/-1 year), menopausal status at diagnosis (premenopausal, postmenopausal, unknown) and factors at one or both blood draws: menopausal status (premenopausal, postmenopausal, unknown), month of collection (+/-1 month), time of day (+/-2 hours), fasting status (>8, ≤8 hours), and postmenopausal hormone use (yes/no). Premenopausal NHSII cases and controls additionally matched on luteal day at blood collection (date of next menstrual cycle minus date of blood draw, +/- 1 day)). All models adjusted for: parity (nulliparous, 1 pregnancy, 2 pregnancies, 3 pregnancies, 4+ pregnancies), OC use (never, <1 year, 1-5 years, 5+ years), tubal ligation (yes, no), marital status (never, married/domestic partnership/living with partner, divorced/separated, widowed), and weight change between ages 18 and blood collection (kg, continuous). | | | | | | | | | | | | | | | | | |
